# Pre- and post-COVID-19 evaluation of antimicrobial susceptibility for healthcare-associated infections in the intensive care unit of a tertiary hospital

**DOI:** 10.1590/0037-8682-0090-2021

**Published:** 2021-07-23

**Authors:** Gilberto Gambero Gaspar, Lécio Rodrigues Ferreira, Cinara Silva Feliciano, Cláudio Penido Campos, Fabiana Murad Rossin Molina, Andrea Cristina Soares Vendruscolo, Giovana Marcão Araújo Bradan, Nátali Artal Padovani Lopes, Roberto Martinez, Valdes Roberto Bollela

**Affiliations:** 1 Universidade de São Paulo, Faculdade de Medicina de Ribeirão Preto, Divisão de Doenças Infecciosas e Tropicais, Departamento de Clínica Médica, Ribeirão Preto, SP, Brasil.; 2 Universidade de São Paulo, Faculdade de Medicina de Ribeirão Preto, Hospital de Clínicas, Serviço de Controle de Infecção, Ribeirão Preto, SP, Brasil.

**Keywords:** COVID-19, Bacterial resistance, Healthcare related infection

## Abstract

**INTRODUCTION:**

Antimicrobial resistance has worsened since the onset of COVID-19.

**METHODS:**

This study involved patients admitted to the adult intensive care unit (ICU) of a tertiary hospital. Pre- and post-COVID-19 data were analyzed. The healthcare-related infections (HCRIs) reported between January 2018 and January 2020 and during the pandemic between February and July 2020 were compared.

**RESULTS:**

Antimicrobial resistance increased during the pandemic, especially for *Klebsiella pneumoniae* isolates, with a rate increase from 5% to 50% for Polymyxin B.

**CONCLUSIONS:**

The susceptibilities of the main pathogens associated with HCRIs in the ICU changed and should be considered in managing severe COVID-19.

The first COVID-19 case in Brazil was diagnosed in February 2020. During the first four months, moderate and severe cases that require hospitalization were reported. Patients usually had comorbidities or were undergoing prolonged immunosuppressive treatment. The most common development was the need for invasive procedures (ex. mechanical ventilation and central access) due to exacerbated inflammatory response mainly after the second week of symptom onset^1^. 

These severe patients can present secondary bacterial or fungal infections during the hospitalization; however, the rate of antibiotic use (94-100%) was usually much higher than the incidence of secondary infections (10-15%). Classically, viral infections lead to an increase in the incidence of bacterial infections; this has been evidenced during major viral epidemics (e.g., pandemic influenza H1N1), but it has been observed to a lesser extent during the SARS-COV-2 epidemic^1^ ^,^ ^2^. Health services were also overburdened, leading to sub-optimal care and, sometimes, the lack of medical supplies^1^.

COVID-19 has led to excessive prescriptions of antimicrobials in the ICU for patients who develop the severe form of the disease, which causes Severe Acute Respiratory Syndrome, even in the absence of a confirmation or strong suspicion of bacterial infection. The WHO highlighted antimicrobial resistance as an invisible pandemic; the increasing death rates, as a result, may culminate in 10 million deaths per year associated with bacterial infections resistant to various classes of antimicrobials by 2050. Thus, the adoption of strategies to restore and strengthen programs for the rational use of antimicrobials in healthcare units has been recommended^3^.

Terni Hospital (Italy) established a program for the rational use of antimicrobials due to the increased incidence of carbapenem-resistant enterobacteriaceae (CRE) in 2016. This program has been maintained and enhanced during the COVID-19 pandemic^4^ ^,^ ^5^ ^,^ ^6^. To control this situation effectively, we must know the local epidemiology of these microorganisms through their phenotypic and genotypic variations (clonal type) and the pharmacokinetic/pharmacodynamic relationship of antimicrobials. All of these endeavors, together with the appropriate measures, will prevent healthcare-related infections^7^.

This was a descriptive, observational, and retrospective study performed at Clinics Hospital Ribeirão Preto School of Medicine (HCRP), São Paulo State, Brazil. The study population included adults admitted to the ICU and transferred to an exclusive COVID-19 ICU just after the onset of the pandemic (February 2020) within the same physical area with the same health professional team. 

The patient data were collected from electronic medical records. The microbiological information was collected at the Microbiology Lab between January 1, 2018, and July 31, 2020. The incidence of infections was estimated by the Hospital Infection Control Committee based on the infection notification criteria by the Health Surveillance Agency of Brazil (ANVISA).

Patients older than 18 years who were admitted to the adult ICU were included. The study period was divided into two: pre-pandemic: data collection from January 1, 2018, to January 31, 2020 (the period before the first case in Brazil); COVID-19 pandemic**:** data collection from February 1 to July 31, 2020 (the period including the month the first case was reported in Brazil).

Antimicrobial susceptibility based on the minimum inhibition concentration (MIC) was determined using the Vitek-2 equipment and breakpoints adopted by the BrCAST (Brazilian Committee for Antimicrobial Susceptibility Testing) guidelines, a document updated on May 20, 2020^8^. The data were descriptively analyzed; the monthly rates of the resistance of isolates (infection or colonization) during the pre-pandemic and the pandemic periods were analyzed. We also compared the incidence of healthcare-related infections during both periods.

We analyzed 466 clinical positive samples for one of the microorganisms studied in a total of 8,408 patients per day, with the following distribution: 2018, 3,007 patients/day; 2019, 3,719 patients/day; and 2020 (until July), 1.682 patients/day. The distribution of the positive cultures showed 246 positive samples for *Klebsiella pneumoniae* (53%), 173 positive samples for *Acinetobacter baumannii* (37%), and 47 positive samples for *Staphylococcus aureus* (10%). The clinical samples isolated from those microorganisms included blood, surgical wound, catheter tip, urine, tracheal secretion, and rectal swab.

Among the resistance rates of the microorganisms studied from January 2018 to July 2020, *Acinetobacter baumannii* had the highest carbapenem resistance rate (78.6%). The rate of *Klebsiella pneumoniae* resistance to polymyxin B increased (15%); when only isolates resistant to carbapenems and polymyxin B were considered, the rate of *Klebsiella pneumoniae* resistance to polymyxin B was 24.1% (Table 1).

**TABLE 1:** Rates of *Staphylococcus aureus* resistance to oxacillin, *Acinetobacter baumannii* resistance to carbapenems, and *Klebsiella pneumoniae* resistance to polymyxin B in an Intensive Care Unit between January 2018 and July 2020 in Ribeirão Preto, SP, Brazil.

Microorganism (total = 446)	Resistance Rate (%)
*Staphylococcus aureus* resistance to oxacillin	35/47 (74,4%)
*Acinetobacter baumannii* resistance to carbapenem	136/173 (78,6%)
*Klebsiella pneumoniae* resistance to carbapenem	153/246 (62,1%)
*Klebsiella pneumoniae* resistance to polimyxin B	37/246 (15,0%)

The rate of *Acinetobacter baumannii* resistance to carbapenems significantly increased in 2020 (35 samples positive for *Acinetobacter baumannii*/1000 patients/day) based on the evaluation of the rates between May and July of 2018, 2019, and 2020 (Figure 1A). 

**FIGURE 1:**
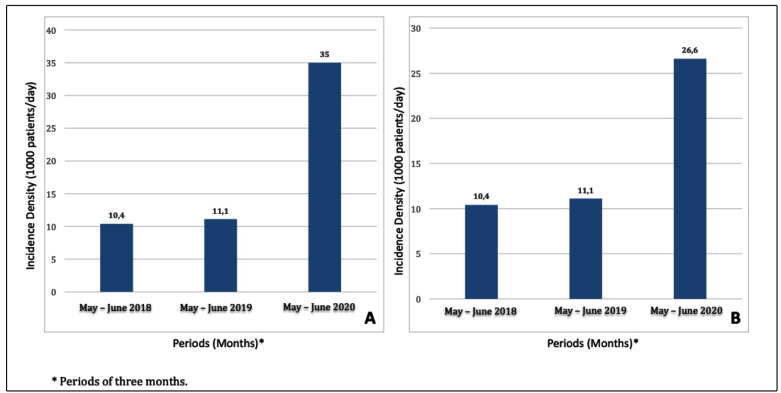
Incidence density (1000 patients/day) for positive samples of carbapenem-resistant *Acinetobacter baumannii* **(A)** and *Klebsiella pneumonia* resistant to polymyxin B **(B)**, from May to July of 2018, 2019 and 2020 for patients admitted to intensive care unit (ICU) at Ribeirão Preto Clinics Hospital, SP, Brazil.

 The incidence density of infections of *Klebsiella pneumoniae* resistant to carbapenems also increased during the pandemic relative to the pre-pandemic period (22 samples positive for *Klebsiella pneumoniae* resistant to carbapenem/1000 patients/day x 15.1 samples positive *for Klebsiella pneumoniae* resistant to carbapenem/1000 patients/day)*.* When the periods between May and July in 2018, 2019, and 2020 were compared, the same trend demonstrated for *Acinetobacter baumannii* was observed for *Klebsiella pneumoniae* in 2020 (26.6 positive samples for *Klebsiella pneumoniae* resistant to carbapenem/1000 patients/day). 

The 37 clinical samples of *Klebsiella pneumoniae* resistant to polymyxin B were categorized based on their sources as follows: tracheal secretion (13; 35%), catheter (7; 19%), urine (5; 13%), blood (5; 13%), rectal swab (4; 10%), and others (10%).

There was also a significant increase in culture positivity for polymyxin B-resistant *Klebsiella pneumoniae* during the pandemic relative to before the pandemic, with prevalence of 50% and 5%, respectively. These isolates were resistant to most antimicrobials tested, except some aminoglycosides that showed a susceptible profile (2; 5%). All of them were susceptible to ceftazidime/avibactam (37; 100%). Most isolates had MICs above 16 mg/dL (above 90%) for polymyxin B.

The incidence density of polymyxin B-resistant *Klebsiella pneumoniae* infections also increased during the pandemic relative to the pre-pandemic period (18.5 positive samples for *Klebsiella pneumoniae* resistant to polymyxin B/1000 patient/day). The incidence density, based on evaluations of the periods between May and July in 2018, 2019, and 2020, increased in 2020 (26.6 positive samples for *Klebsiella pneumoniae* resistant to polymyxin B/1000 patient/day) (Figure 1B).

Ventilator-associated pneumonia (VAP) was the main infection during the pandemic period. Based on the outcomes of this study, COVID-19 significantly increased the rates of healthcare-related infection (mainly VAP) in the intensive care units for COVID-19. We also observed an increase in the number of microorganisms resistant to various antimicrobials, especially polymyxin B-resistant *Klebsiella pneumoniae*. Considering this worrying scenario, multi-drug resistance, together with the limited therapeutic arsenal, may be among the main contributors to mortality in patients with antibiotic-resistant infections. This should serve as a trigger for the promotion of preventive measures for the rational use of antimicrobials and multidisciplinary strategies for the prevention of healthcare-related infections.
